# Emotion Dysregulation Moderates the Association Between Stress and Problematic Cannabis Use

**DOI:** 10.3389/fpsyt.2020.597789

**Published:** 2021-01-08

**Authors:** Jessica M. Cavalli, Anita Cservenka

**Affiliations:** Substance Use and Neurocognition Lab, School of Psychological Science, Oregon State University, Corvallis, OR, United States

**Keywords:** cannabis, cannabis problems, emotion dysregulation, stressful life events, perceived stress

## Abstract

**Background:** Research suggests emotion dysregulation is a transdiagnostic risk factor for substance use and addiction and that stress may lead to problematic cannabis use. Thus, the current study examines how emotion dysregulation moderates the associations between stress (stressful life events and perceived stress) and problematic cannabis use.

**Methods:** Eight hundred and fifty-two adults reporting any lifetime cannabis use completed an anonymous online survey. Participants completed a brief demographic questionnaire and were asked to report their past 30-day use of cannabis, alcohol, nicotine, and illicit substances. Problematic cannabis use (*via* the Marijuana Problem Scale), emotion dysregulation (*via* the Difficulties in Emotion Regulation Scale), perceived stress (*via* the Perceived Stress Scale), and stressful life events (*via* the Holmes-Rahe Life Stress Inventory) were assessed. Hierarchical multiple linear regressions were conducted.

**Results:** Findings indicate that when examining the moderating role of emotion dysregulation, more stressful life events and less perceived stress were associated with more severe problematic cannabis use, and these associations were stronger at higher levels of emotion dysregulation.

**Conclusions:** These results demonstrate a strong step toward understanding how emotion dysregulation moderates the relationship between stress and problematic cannabis use; however, longitudinal studies are needed to determine directionality of effects. Overall, these results suggest the importance of examining emotion dysregulation as a moderator of both stressful life events and stress perception as they relate to problematic cannabis use.

## Introduction

Over the past several decades, cannabis has been reported as the most commonly used illicit substance in the United States (1). As legalization for medical and recreational cannabis has been expanding, perceived risk of using cannabis has been steadily declining since the late 1980's, while prevalence of use has been steadily increasing (2, 3). These trends persist despite growing evidence that cannabis use is associated with adverse health and life outcomes, such as addiction, mental health issues, and cognitive impairment (4–6). Thus, determining factors that influence the escalation toward problematic cannabis use is critical for understanding how to effectively implement prevention, intervention, and treatment efforts. Research suggests that emotion dysregulation is a transdiagnostic risk factor for substance use and addiction (7–9). Moreover, using cannabis to cope with stress and negative affect may lead to greater cannabis use-related problems and cannabis use disorder; thus, additional studies examining how individual differences in emotion dysregulation may affect these relationships are crucial for understanding who may be at a higher risk for experiencing problematic cannabis use (10).

Cannabis contains over 100 cannabinoids, including Δ9-tetrahydrocannabinol (Δ9-THC), the main psychoactive component of cannabis, and cannabidiol (CBD), a non-psychoactive component, which exert their effects by modulating the endocannabinoid system (ECS) at cannabinoid receptor types 1 and 2 [CB1 and CB2; (11, 12)]. CB1 receptors are expressed in distinct neuronal subpopulations in the forebrain, with high levels on GABAergic interneurons and lower levels on glutamatergic neurons and their activation leads to an inhibitory effect on GABA and glutamate release (11, 13). Differential involvement of CB1 receptors at GABAergic and glutamatergic neurons have been shown to influence social behaviors, fear, anxiety and feelings of reward and aversion (14–17). Further, research suggests that there is cross-talk between dopaminergic receptors and the ECS regarding the control of negative affect, anxious behaviors, reward, and aversion (14, 17, 18).

Accumulating evidence suggests that the ECS is involved in the neural modulation of the stress response, *via* regulation of the hypothalamic-pituitary-adrenal (HPA) axis, the major neuroendocrine system responsible for the stress response [see (19) for a review]. High densities of CB1 receptors are present in several limbic brain regions, including the hippocampus, amygdala, and prefrontal cortex, that are involved in HPA axis regulation and the interpretation of psychological stressors (19). Moreover, alterations in the ECS are found in several psychopathologies, especially those with emotion-related dysfunction as a core symptom (14). Pre-clinical and clinical research shows a strong association between the hypofunction of endocannabinoid signaling and mood disorders, such as depression (12, 20). In contrast, the facilitation of endocannabinoid signaling *via* CB1 and/or CB2 receptor activation produces similar effects of current antidepressants, which may be a therapeutic avenue in treating major depression and other affective-related disorders (20). Therefore, given the involvement of endocannabinoid signaling in stress and emotional processes, and the influence of cannabis on the ECS, it is relevant to examine these processes in cannabis users.

Stressful life events, such as childhood adversities, family dysfunction, social disadvantage, trauma, and other negative life events may put individuals at risk for early onset cannabis use, greater coping-motivated use, lifetime cannabis use, and cannabis use disorder (21–23). Stressful life events are associated with recent cannabis use, greater odds of women's cannabis use during the perinatal period, and the maintenance of cannabis use across adolescent development into adulthood (24–26). Furthermore, past year stressful life events predict the transition from frequent cannabis use to cannabis dependence (27). These studies suggest that experiencing more stressful life events may put an individual at risk for greater cannabis use, more cannabis use-related problems, and an increased risk for cannabis use disorder. Though, because individuals may experience the same stressful life event differently, it is also important to consider how perceived stress may relate to problematic cannabis use.

Previous studies indicate greater perceived stress to be associated with more problematic cannabis use (28–30). For example, Spradlin and Cuttler (30) found that among college students, perceived stress was significantly associated with experiencing more cannabis-use related problems. Further, this relationship was also mediated by coping motives, suggesting that these individuals may be using cannabis to cope with their perceived stress. Though, it is possible that a more complex relationship exists beyond just perceived stress and problematic cannabis use. Ketcherside and Filbey (28) found that among current heavy cannabis users, perceived stress was significantly associated with more problematic cannabis use, which was mediated by depression and anxiety. These findings indicate that the role of negative affect should also be considered when investigating the relationship between perceived stress and problematic cannabis use. Thus, to further understand the association between stress and problematic cannabis use, it is important to investigate individual differences that could moderate this relationship, such as emotion regulation, which is one potential key ability that can reduce stress symptoms and buffer the negative effects of stress (31–34).

Several studies report that cannabis users experience emotion dysregulation and that emotion dysregulation is associated with cannabis use outcomes, such as higher cannabis consumption, cannabis abuse, and problematic cannabis use (35–38). Research suggests that cannabis users with greater emotion dysregulation are more apt to use cannabis as a coping mechanism (39, 40). Moreover, using cannabis as a coping mechanism for stress and negative affect has been found to mediate the relationship between emotion dysregulation and problematic cannabis use (41). Therefore, if individuals experiencing greater stress use cannabis to cope with stress and regulate negative affect, these individuals may be at risk of problematic cannabis use. Finally, emotion dysregulation mediates the relationship between depression, anxiety, and suicidal ideation and problematic cannabis use, further suggesting that a pathway to problematic use could be through an inability to effectively regulate negative emotions (42). Overall, research suggests that the development of problematic cannabis use could be attributed to users' emotion dysregulation, and a need to better regulate negative affect when under stressful conditions.

Since the impact of stress on problematic substance use may be attenuated in individuals who can better regulate their emotions during stressful events (34), emotion dysregulation may be an important moderator variable when considering the relationship between stress and problematic cannabis use. In fact, several studies support emotion dysregulation as an important moderator variable between stress and substance use outcomes, such as cannabis dependence, alcohol use, adolescent substance use, and substance use severity among women experiencing post-traumatic stress symptoms (9, 43–45). However, no known research has examined the moderating role of emotion dysregulation in the association between stress and problematic cannabis use. Therefore, it is critical to examine how these interactions found between emotion dysregulation and stress may extend to problematic cannabis use. Examining emotion dysregulation as moderator could determine whether individual differences in emotion dysregulation may exacerbate the effect of stress on problematic cannabis use. As such, the primary aim of the current study was to test the role of emotion dysregulation as a moderator for the relationship between stress and problematic cannabis use.

Most research that examines stress and cannabis use rarely directly compares how different types of stress are related to problematic cannabis use (24–27, 29). Thus, there is a significant gap in our understanding of the nature of the association between stress and problematic cannabis use. Because stressful life events and perceived stress measure different aspects of stress, it is important to consider how they are each individually related to problematic cannabis use (46). In order to thoroughly examine our primary aim, we assessed two types of stress (past year stressful life events and perceived stress) to better understand how one type of stress relates to problematic cannabis use, while accounting for the other. Thereupon, we address an important gap in our understanding of the types of stress that may be risk factors for problematic cannabis use. We predicted that more stressful life events, greater perceived stress, and greater emotion dysregulation would be associated with greater problematic cannabis use. Further, we hypothesized that the relationship between stress and problematic cannabis use would be moderated by emotion dysregulation, such that it would be stronger in individuals with greater emotion dysregulation.

## Materials and Methods

### Participants

See Table 1 for a complete description of sample demographics. After data cleaning (see Supplementary Material), the final sample included 852 cannabis users. One participant was excluded because they were the only individual to report “Other” as their biological sex, and sex was ultimately chosen as a covariate for analyses (see analytic plan in section Data Analysis). The sample was on average 26.88 (SD = 6.71) years old, predominately white (65.5%), male (63%), middle class (61.5%), not Spanish/Hispanic/Latinx (67.8%), and had completed some college (35.6%). The majority of participants had last used cannabis <1 month ago (68.2%), used cannabis 2+ days/week on average in the past year (58%), and used cannabis on more than 100 days in their lifetime (52.1%). While approximately a third of the sample used more than once per day on weekdays (32.6%), nearly one half of the sample used more than once per day on weekends (46.3%).

**Table 1:** Demographics, substance use characteristics, and scores on primary variables.

**Demographics (*N* = 852)**	***M* (SD) or %**
Age	26.88 (6.71)
Sex (% Male)	63.0
Ethnicity	
Hispanic/latinx	32.20
Not hispanic/latinx	66.80
Unknown	1.00
Race	
White	65.60
Black or African American	19.10
American Indian or Alaskan Native	4.60
Asian	3.80
More than one race	3.50
Native Hawaiian/Other Pacific Islander	2.00
Other	1.20
Unknown	0.20
Income	
$0	1.10
$0–$5,000	2.70
$5,000–$10,000	5.60
$10,000–$50,000	16.90
$50,000–$75,000	41.90
$75,000–$100,000	19.60
>$100,000	12.20
Highest level of education	
Some high school	0.40
High school diploma/GED	6.20
Trade/technical/vocational training	8.50
Some college	35.60
Associate's degree	14.70
Bachelor's degree	27.80
Some graduate school	4.80
Graduate school or professional degree	2.10
**Past 30-day substance use (*****N*** **=** **852)**	
Cannabis use days^a^	9.46 (8.51)
Alcoholic drinks^b^	6.89 (14.59)
Cigarettes^c^	10.66 (93.46)
E-Nicotine use days^d^	1.40 (4.87)
Illicit substances^e^	0.14 (1.08)
**Scores on predictor and outcome variables (*****N*** **=** **852)**	
MPS total score	12.76 (10.12)
DERS total score	97.56 (19.93)
H-RLSI total score	150.49 (112.84)
PSS total score	26.71 (5.68)

*Scores on the Marijuana Problem Scale (MPS) are between 0 and 38. Scores on the Difficulties in Emotion Regulation Scale (DERS) are between 36 and 180. Scores on the Holmes-Rahe Life Stress Inventory (H-RLSI) are between 0 and 1,466. Scores on the Perceived Stress Scale (PSS) are between 0 and 56*.

a *Number of days cannabis was used in the past 30 days*.

b *Number of drinks consumed in the past 30 days*.

c *Number of cigarettes consumed in the past 30 days*.

d *Number of days e-nicotine products were used in the past 30 days*.

e *Number of times illicit substances other than cannabis products were used in the past 30 days*.

Participants were recruited through community flyers and word of mouth. Additionally, students attending a Pacific Northwest university were able to access the survey through SONA, the university's research subject pool, in order to gain credit toward courses in which they were enrolled. While these recruitment efforts took place in Oregon, it is likely that participants outside of Oregon completed the survey, since the survey was also advertised on multiple social media platforms, allowing it to be shared across states lines *via* snowball sampling. At the request of the Oregon State University Institutional Review Board, university student status and participants' state of residence were not collected in order to maintain participant anonymity. Eligible participants were U.S. citizens who were age of majority and fluent in the English language.

### Procedure

Participants completed the ~60-min survey through an anonymous Qualtrics link. SONA participants were compensated with research credit for their classes. Non-SONA participants were compensated with an electronic $5 Amazon gift card and entered into a raffle to win an electronic $100 Amazon gift card. All procedures were in accordance with the guidelines of the Oregon State University Institutional Review Board.

### Measures

Participants filled out a brief demographic questionnaire that included multiple choice questions on biological sex, income, highest level of education, race, and ethnicity. Participants completed the Daily Sessions, Frequency, Age of Onset, and Quantity of Cannabis Use Inventory [DFAQ-CU; (47)]. Previous research has established the factor structure, reliability, and validity of this measure (47). In order to control for any effect of recent cannabis use, we used the question in the DFAQ-CU on the number of days cannabis was used in the past 30 days. Participants also reported their past 30-day use of alcohol, nicotine, and illicit substances.

Participants completed the Marijuana Problem Scale [MPS; (48)] to assess their severity of problematic cannabis use. Previous research has established the internal reliability of this measure (48–50). This measure includes 19 questions that ask about negative consequences related to one's cannabis use in the past month. They are rated as no problem (0), minor problem (1), or serious problem (2) and there are two ways to score this measure: with the total problem score (i.e., number of items scored as minor or serious, 0–19) and with a total severity score [i.e. summed score across the 19 items indicating severity rating 0–38; (50)]. Previous research has shown that the total severity score performs slightly better on a psychometric evaluation compared to the total problem score (50). Moreover, the total severity score has been used in previous research examining associations between stress and problematic cannabis use (30). Finally, our aim was to investigate how a more nuanced measure of seriousness of cannabis-related problems relates to stress and emotion dysregulation. Thus, the total MPS severity score was used as the dependent variable in the main aims of the current study.

The Difficulties in Emotion Regulation Scale was used to assess participants' self-reported ability to regulate their emotions [DERS; (51)]. Previous research has found this measure to have high internal consistency, good test-retest reliability, and adequate construct and predictive validity (51). This measure includes 36 items that ask how often statements regarding participants' ability to regulate emotion apply to them. They are rated as almost never (1), sometimes (2), about half the time (3), most of the time (4), and almost always (5). The total score was used as an independent variable in the analytical plan described in section Data Analysis.

Participants completed the Holmes-Rahe Life Stress Inventory [H-RLSI; (52)]. The H-RLSI includes a list of stressful life events and participants are instructed to endorse the events they have experienced in the past year. Previous research has established the reliability and validity of this measure (52, 53). There are 43 different life events, each with a different point value (e.g., death of a spouse = 100, minor violations of the law = 11). The total sum score was used as the independent variable in the analytical plan described in section Data Analysis.

Participants also completed the Perceived Stress Scale [PSS; (54)]. The PSS includes statements to determine participants' perceived stress in the past month. Previous research has established the reliability and validity of this measure (54, 55). There are 14 questions that ask participants the degree to which situations in one's life are appraised as stressful. They are rated as never (0), almost never (1), sometimes (2), fairly often (3), and very often (4). The total score was used as the independent variable in the analytical plan described in section Data Analysis.

### Data Analysis

For all statistical analyses, SPSS Version 26.0 (56) was used and alpha was set to 0.05. Because assumptions of linear regression were met, parametric tests were used for all analyses. To assess emotion dysregulation as a moderator between stress and problematic cannabis use, we used hierarchical multiple linear regression. Correlation analysis (Pearson's *r*) was used to examine the relationships between emotion dysregulation (*via* the DERS) and problematic cannabis use (*via* the MPS), stressful life events (*via* the H-RLSI) and MPS, and perceived stress (*via* the PSS) and MPS.

Further, to determine covariates to include in our regression models, correlation analysis, independent samples *t*-test, and analysis of variance were used to assess the possible relationships between our primary variables of interest, demographic variables, and substance use variables (see Table 2). The first two hierarchical multiple linear regression models (Models 1 and 2) were primary analyses; these included the six demographic variables (age, biological sex, race, ethnicity, income, and highest level of education), past 30-day cannabis use, and either stressful life events or perceived stress as covariates. The second two hierarchical multiple linear regression models (Models 3 and 4) served as secondary analyses and also included past 30-day alcoholic drinks and past 30-day e-nicotine use as covariates; the purpose of these secondary analyses were to help identify whether emotion dysregulation moderated the association between stress and problematic cannabis use above and beyond co-occurring alcohol and e-nicotine use in this sample. The results of Models 3 and 4 can be found in Supplementary Material.

**Table 2:** Average scores, groups differences, and correlations with the marijuana problem scale.

**Demographics (*N* = 852)**	***M* (SD)**	***r, t*, or *F***	***p***
Age		*r* = 0.29	** <0.001**
Sex		12.52	** <0.001**
Male	15.82 (9.77)		
Female	7.56 (8.46)		
Ethnicity		36.10	** <0.001**
Hispanic/latinx	16.84 (9.36)		
Not hispanic/latinx	10.87 (9.91)		
Unknown	7.78 (8.71)		
Race		17.07	** <0.001**
Native Hawaiian/Other Pacific Islander	18 (7.97)		
Black or African American	17.36 (8.60)		
American Indian or Alaskan Native	16.51 (8.37)		
White	12.20 (10.31)		
Other	5.30 (5.72)		
More than one race	3.53 (4.36)		
Asian	3.44 (3.88)		
Unknown	1.00 (1.41)		
Income		33.41	** <0.001**
0.00	0.78 (1.20)		
$0–$5,000	3.17 (3.97)		
$5,000–$10,000	5.04 (6.71)		
$10,000–$50,000	11.69 (8.74)		
$50,000–$75,000	17.04 (9.94)		
$75,000–$100,000	12.41 (9.37)		
>$100,000	6.80 (8.42)		
Highest level of education		39.28	** <0.001**
Some high school	17.33 (1.53)		
High school diploma/GED	5.66 (7.45)		
Trade/technical/vocational training	13.92 (6.36)		
Some college	8.07 (8.41)		
Associate's degree	12.50 (9.47)		
Bachelor's degree	19.59 (10.02)		
Some graduate school	16.85 (7.07)		
Graduate school or professional degree	9.61 (10.16)		
**Past 30-day substance use (*****N*** **=** **852)**			
Cannabis use days^a^		*r* = 0.08	**0.03**
Alcoholic drinks^b^		*r* = −0.31	** <0.001**
Cigarettes^c^		*r* = −0.03	0.33
E-Nicotine use days^d^		*r =* −0.17	** <0.001**
Illicit substances^e^		*r <*0.01	0.96

*Bold p-values indicate significant group differences on the Marijuana Problem Scale (MPS) or significant correlations with scores on the MPS*.

a *Number of days cannabis was used in the past 30 days*.

b *Number of drinks consumed in the past 30 days*.

c *Number of cigarettes consumed in the past 30 days*.

d *Number of days e-nicotine products were used in the past 30 days*.

e *Number of times illicit substances other than cannabis products were used in the past 30 days*.

We used hierarchical multiple linear regression to assess whether emotion dysregulation (*via* the DERS), stressful life events (*via* the H-RLSI), and their interaction were related to problematic cannabis use (*via* the MPS). Perceived stress was included in this model as a covariate. Covariates were entered into the first block of the regression to control for these variables. The DERS and H-RLSI were mean-centered and added into the second block to assess whether they were significantly associated with scores on the MPS after controlling for covariates entered in block 1. Finally, the interaction term for the DERS and H-RLSI was added into the third block to assess whether it was significantly related to scores on the MPS above and beyond the main effects and covariates.

Similarly, we also used hierarchical multiple linear regression to assess whether emotion dysregulation (*via* the DERS), perceived stress (*via* the PSS), and their interaction were significantly associated with problematic cannabis use (*via* the MPS). For this model, stressful life events was included as a covariate. We included the same covariates for Models 3 and 4, as were included in Models 1 and 2, respectively.

## Results

Pearson's correlations revealed that emotion dysregulation (*r* = 0.53, *p* < 0.001), stressful life events (*r* = 0.32, *p* < 0.001), and perceived stress (*r* = 0.13, *p* < 0.001) were significantly, positively correlated with problematic cannabis use (Table 3). To evaluate for multicollinearity, Pearson's correlations were used between scores on the DERS, PSS, and H-RLSI and an *a priori* level of <0.70 was established to determine whether these constructs were relatively independent measures (57); all correlations were below this cut-off. Moreover, analysis of collinearity statistics showed this assumption was met, as Variance Inflation Factor scores were well below 10, and tolerance scores above 0.2 (statistics were on average 2 and 0.6, respectively).

**Table 3:** Correlations (Pearson's *r*) between primary variables.

	**1**	**2**	**3**	**4**
1. MPS	1			
2. H-RLSI	0.32	1		
3. PSS	0.13	0.13	1	
4. DERS	0.53	0.17	0.53	1

*All p's < 0.001. MPS, Marijuana Problem Scale; H-RLSI, Holmes-Rahe Life Stress Inventory; PSS, Perceived Stress Scale; DERS, Difficulties in Emotion Regulation Scale*.

**Figure 1:**
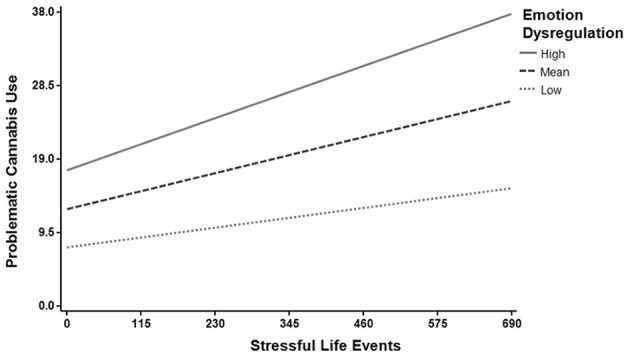
Emotion dysregulation moderates the relationship between stressful life events and problematic cannabis use. The relationship between stressful life events and problematic cannabis use strengthens as emotion dysregulation increases. The moderating role of emotion dysregulation was tested at the average score (Mean), and one standard deviation above (High) and below (Low) the mean.

Regression Model 1 tested whether stressful life events, emotion dysregulation, and their interaction were related to problematic cannabis use. We found a significant regression equation [*F*_(29, 822)_ = 43.12, *p* < 0.001] with *R*^2^ = 0.60 (see Table 4 for the model summary). Emotion dysregulation (*B* = 0.42, *p* < 0.001), stressful life events (*B* = 0.20, *p* < 0.001) and their interaction (*B* = 0.05, *p* = 0.04) were significantly associated with problematic cannabis use (see Table 5 for all coefficients in the model). As Figure 1 shows, the relationship between stressful life events and problematic cannabis use strengthens as emotion dysregulation increases. To probe the interaction, simple slopes analysis was used by looking one standard deviation above and below the mean for emotion dysregulation. The relationship between stressful life events and problematic cannabis use remained significant at both high levels of emotion dysregulation (*B* = 0.27, *p* < 0.001) and low levels of emotion dysregulation (*B* = 0.15, *p* < 0.001).

**Table 4:** Model summary for model 1: effects of emotion dysregulation, stressful life events, and their interaction on problematic cannabis use.

**Model summary**										
	***R***	***R***^**2**^	**Adj**. ***R***^**2**^	**Std. error of the estimate**	***R***^**2**^ **change**	***F*** **change**	**df1**	**df2**	**Sig**. ***F*** **change**	**Cohen's** ***f***^**2**^
Block 1	0.67	0.45	0.44	7.60	0.45	26.21	26	825	** <0.001**	
Block 2	0.76	0.60	0.59	6.60	0.15	153.50	2	823	** <0.001**	0.38
Block 3	0.78	0.60	0.59	6.48	0.002	4.42	1	822	**0.04**	0.008

*Bold p-values indicate significant F change*.

**Table 5:** Coefficients for model 1: effects of emotion dysregulation, stressful life events, and their interaction on problematic cannabis use.

**Coefficients**											
		**Unstandardized**		**Standardized**			**95% CI for** ***B***		**Correlations**		
		***B***	**Std. error**	**Beta**	***t***	**Sig**.	**Lower bound**	**Upper bound**	**Zero-order**	**Partial**	**Part**
Block 1	(Constant)	5.83	3.18		1.84	0.07	−0.40	12.06			
	Age	0.05	0.04	0.04	1.29	0.20	−0.03	0.13	0.29	0.05	0.03
	Sex	−2.36	0.53	−0.11	−4.45	** <0.001**	−3.40	−1.32	−0.39	−0.15	−0.10
	Hispanic/latinx	2.51	0.55	0.12	4.59	** <0.001**	1.43	3.58	0.28	0.16	0.10
	Unknown (hispanic/latinx)	0.61	2.27	0.01	0.27	0.79	−3.84	5.06	−0.05	0.01	0.01
	White	0.46	2.20	0.02	0.21	0.83	−3.86	4.78	−0.08	0.01	0.00
	Black or African American	3.21	2.28	0.12	1.41	0.16	−1.27	7.70	0.22	0.05	0.03
	American Indian or Alaskan Native	5.06	2.47	0.10	2.04	**0.04**	0.20	9.91	0.08	0.07	0.05
	Asian	−3.87	2.50	−0.07	−1.55	0.12	−8.79	1.04	−0.18	−0.05	−0.03
	More than one race	−3.03	2.47	−0.06	−1.23	0.22	−7.88	1.82	−0.17	−0.04	−0.03
	Native Hawaiian/Other Pacific Islander	5.03	2.78	0.07	1.81	0.07	−0.43	10.50	0.07	0.06	0.04
	Unknown (race)	−4.02	5.09	−0.02	−0.79	0.43	−14.00	5.97	−0.06	−0.03	−0.02
	Some high school	6.43	4.21	0.04	1.53	0.13	−1.84	14.69	0.03	0.05	0.03
	High school diploma/GED	−0.66	1.95	−0.02	−0.34	0.73	−4.49	3.16	−0.18	−0.01	−0.01
	Trade/technical/vocational training	0.51	1.89	0.01	0.27	0.79	−3.19	4.22	0.03	0.01	0.01
	Some college	−0.23	1.73	−0.01	−0.13	0.90	−3.62	3.16	−0.34	0.00	0.00
	Associate's degree	1.33	1.73	0.05	0.77	0.44	−2.08	4.73	−0.01	0.03	0.02
	Bachelor's degree	5.04	1.70	0.22	2.97	**0.003**	1.71	8.38	0.42	0.10	0.07
	Some graduate school	3.15	1.93	0.07	1.63	0.10	−0.65	6.94	0.09	0.06	0.04
	$0	0.29	2.31	0.00	0.12	0.90	−4.25	4.82	−0.12	0.00	0.00
	$0–$5,000	−0.60	1.54	−0.01	−0.39	0.70	−3.63	2.43	−0.16	−0.01	−0.01
	$5,000–$10,000	1.31	1.19	0.03	1.10	0.27	−1.02	3.65	−0.19	0.04	0.02
	$10,000–$50,000	4.10	0.89	0.15	4.60	** <0.001**	2.35	5.86	−0.05	0.16	0.10
	$50,000–$75,000	4.66	0.79	0.23	5.91	** <0.001**	3.11	6.21	0.36	0.20	0.13
	$75,000–$100,000	1.39	0.85	0.05	1.63	0.10	−0.28	3.07	−0.02	0.06	0.04
	Cannabis use days^a^	−0.02	0.03	−0.02	−0.74	0.46	−0.08	0.03	0.08	−0.03	−0.02
	Centered PSS scores	−0.28	0.05	−0.15	−5.66	** <0.001**	−0.37	−0.18	0.13	−0.19	−0.12
Block 2	Centered DERS scores	0.21	0.02	0.41	13.85	** <0.001**	0.18	0.24	0.53	0.44	0.31
	Centered H-RLSI scores	0.02	0.00	0.21	8.48	** <0.001**	0.01	0.02	0.32	0.28	0.19
Block 3	DERS X H-RLSI	0.00	0.00	0.06	2.55	**0.01**	0.00	0.00	0.15	0.09	0.06

*PSS, Perceived Stress Scale; DERS, Difficulties in Emotion Regulation Scale; H-RLSI, Holmes-Rahe Life Stress Inventory*.

a *Number of days cannabis was used in the past 30 days*.

*Bold p-values indicate significant coefficient statistics*.

**Figure 2:**
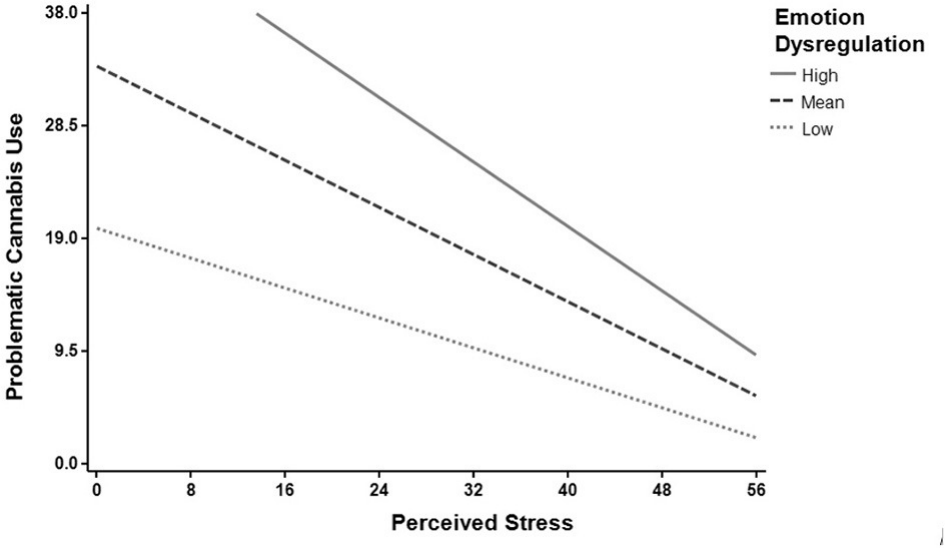
Emotion dysregulation moderates the relationship between perceived stress and problematic cannabis use. The relationship between perceived stress and problematic cannabis use weakens as emotion dysregulation decreases. The moderating role of emotion dysregulation was tested at the average score (Mean), and one standard deviation above (High) and below (Low) the mean.

Regression Model 2 tested whether perceived stress, emotion dysregulation, and their interaction were related to problematic cannabis use. We found a significant regression equation [*F*_(29, 822)_ = 43.35, *p* < 0.001] with *R*^2^ = 0.61 (see Table 6 for the model summary). Emotion dysregulation (*B* = 0.41, *p* < 0.001), perceived stress (*B* = −0.20, *p* < 0.001) and their interaction (*B* = −0.10, *p* = 0.007) were significantly associated with problematic cannabis use (see Table 7 for all coefficients in the model). As Figure 2 shows, the relationship between perceived stress and problematic cannabis use weakens as emotion dysregulation decreases. To probe the interaction, simple slopes analysis was used by looking one standard deviation above and below the mean for emotion dysregulation. The relationship between perceived stress and problematic cannabis use remained significant at both high levels of emotion dysregulation (*B* = −0.24, *p* < 0.001) and low levels of emotion dysregulation (*B* = −0.15, *p* < 0.001). Specifically, lower levels of perceived stress were related to greater problematic cannabis use, but the relationship was weaker for individuals reporting less emotion dysregulation.

**Table 6:** Model summary for model 2: effects of emotion dysregulation, perceived stress, and their interaction on problematic cannabis use.

**Model summary**										
	***R***	***R***^**2**^	**Adj**. ***R***^**2**^	**Std. error of the estimate**	***R***^**2**^ **change**	***F*** **change**	**df1**	**df2**	**Sig**. ***F*** **change**	**Cohen's** ***f***^**2**^
Block 1	0.71	0.50	0.49	7.24	0.50	32.19	26	825	** <0.001**	
Block 2	0.78	0.60	0.59	6.50	0.10	100.68	2	823	** <0.001**	0.25
Block 3	0.78	0.61	0.59	6.50	0.003	7.30	1	822	**0.007**	0.03

*Bold p-values indicate significant F change*.

**Table 7:** Coefficients for model 2: effects of emotion dysregulation, perceived stress, and their interaction on problematic cannabis use.

**Coefficients**											
		**Unstandardized**		**Standardized**			**95% CI for** ***B***		**Correlations**		
		***B***	**Std. error**	**Beta**	***t***	**Sig**.	**Lower bound**	**Upper bound**	**Zero-order**	**Partial**	**Part**
Block 1	(Constant)	6.06	3.17		1.91	0.06	−0.17	12.29			
	Age	0.07	0.04	0.05	1.65	0.1	−0.01	0.15	0.29	0.06	0.04
	Sex	−2.17	0.53	−0.1	−4.07	** <0.001**	−3.22	−1.12	−0.39	−0.14	−0.09
	Hispanic/latinx	2.46	0.55	0.11	4.5	** <0.001**	1.39	3.53	0.28	0.16	0.1
	Unknown (hispanic/latinx)	0.69	2.27	0.01	0.3	0.76	−3.76	5.14	−0.05	0.01	0.01
	White	0.48	2.2	0.02	0.22	0.83	−3.84	4.8	−0.08	0.01	0
	Black or African American	3.24	2.28	0.13	1.42	0.16	−1.24	7.72	0.22	0.05	0.03
	American Indian or Alaskan Native	5.04	2.47	0.1	2.04	**0.04**	0.19	9.89	0.08	0.07	0.04
	Asian	−3.85	2.5	−0.07	−1.54	0.12	−8.76	1.06	−0.18	−0.05	−0.03
	More than one race	−3.25	2.46	−0.06	−1.32	0.19	−8.08	1.59	−0.17	−0.05	−0.03
	Native Hawaiian/Other Pacific Islander	5.15	2.78	0.07	1.85	0.06	−0.32	10.61	0.07	0.06	0.04
	Unknown (race)	−3.12	5.09	−0.01	−0.61	0.54	−13.11	6.86	−0.06	−0.02	−0.01
	Some high school	5.56	4.21	0.03	1.32	0.19	−2.71	13.83	0.03	0.05	0.03
	High school diploma/GED	−0.73	1.95	−0.02	−0.38	0.71	−4.56	3.09	−0.18	−0.01	−0.01
	Trade/technical/vocational training	0	1.89	0	0	.99	−3.71	3.72	0.03	0	0
	Some college	−0.52	1.73	−0.02	−0.3	0.76	−3.92	2.88	−0.34	−0.01	−0.01
	Associate's degree	1.15	1.74	0.04	0.66	0.51	−2.25	4.56	−0.01	0.02	0.01
	Bachelor's degree	4.67	1.71	0.21	2.74	**0.01**	1.32	8.02	0.42	0.09	0.06
	Some graduate school	2.82	1.94	0.06	1.46	0.15	−0.98	6.63	0.09	0.05	0.03
	$0	0.28	2.31	0	0.12	0.9	−4.25	4.82	−0.12	0	0
	$0–$5,000	−0.53	1.54	−0.01	−0.35	0.73	−3.56	2.49	−0.16	−0.01	−0.01
	$5,000–$10,000	1.03	1.19	0.02	0.87	0.39	−1.3	3.36	−0.19	0.03	0.02
	$10,000–$50,000	3.87	0.89	0.14	4.34	** <0.001**	2.12	5.62	−0.05	0.15	0.1
	$50,000–$75,000	4.51	0.79	0.22	5.7	** <0.001**	2.95	6.06	0.36	0.19	0.13
	$75,000–$100,000	1.48	0.85	0.06	1.74	0.08	−0.19	3.15	−0.02	0.06	0.04
	Cannabis use days^a^	−0.02	0.03	−0.01	−0.6	0.55	−0.07	0.04	0.08	−0.02	−0.01
	Centered H-RLSI scores	0.02	0	0.22	8.89	** <0.001**	0.02	0.02	0.32	0.3	0.2
Block 2	Centered DERS scores	0.21	0.02	0.4	13.56	** <0.001**	0.18	0.23	0.53	0.43	0.3
	Centered PSS scores	−0.34	0.05	−0.19	−6.46	** <0.001**	−0.45	−0.24	0.13	−0.22	−0.14
Block 3	DERS X PSS	0	0	−0.07	−2.61	**0.01**	−0.01	0	−0.26	−0.09	−0.06

*H-RLSI, Holmes-Rahe Life Stress Inventory; DERS, Difficulties in Emotion Regulation Scale; PSS, Perceived Stress Scale*.

a *Number of days cannabis was used in the past 30 days*.

*Bold p-values indicate significant coefficient statistics*.

After including past 30-day alcoholic drinks and past 30-day e-nicotine use as covariates for Models 3 and 4, results remained similar between Models 1 and 3 (examining stressful life events; Supplementary Tables 1, 2) and between Models 2 and 4 (examining perceived stress; Supplementary Tables 3, 4).

## Discussion

The current study investigated whether emotion dysregulation and stress were associated with problematic cannabis use, and if individual differences in emotion dysregulation moderated the relationship between stress and problematic cannabis use. The main findings of the current study indicated that emotion dysregulation moderated the relationship between stressful life events and problematic use, such that greater stressful life events were related to more severe problematic cannabis use, and this relationship was stronger in individuals with greater emotion dysregulation. Thus, individuals with difficulties regulating emotions and who experience greater stressful life events may be among those at highest risk for developing problematic cannabis use.

When examining emotion dysregulation as a moderator of the relationship between perceived stress and cannabis use, it was found that at low levels of perceived stress, problematic cannabis use was highest for those with greater emotion dysregulation. However, at high levels of perceived stress individual differences in emotion regulation capacity had a weaker effect on the association between perceived stress and problematic cannabis use. Thus, once the shared variance between perceived stress and emotion dysregulation was accounted for, perceived stress was negatively related to problematic cannabis use. These findings indicate an interesting example of cross-over suppression and support the importance of examining emotion dysregulation and perceived stress in the same model (58, 59). In fact, this relationship is opposite to what was found for the model that included stressful life events, emotion dysregulation, and their interaction as independent variables, further suggesting that different measures used to assess stress, especially those that are relatively weakly correlated (*r* = 0.13 for PSS and H-RSLI) can relate to problematic cannabis use in unique ways when emotion dysregulation is included in these models.

The findings from the second model described above suggest that cannabis users experiencing lower perceived stress, but greater emotion dysregulation, experience more severe problematic cannabis use. While speculative, individuals higher in emotion dysregulation may use cannabis to achieve lower perceived stress, and the HPA axis may play a role in this response. Chronic cannabis use may result in blunted HPA axis reactivity and dampened emotional reactivity to stress at both physiological and psychological levels (60, 61), which is in line with the role of the ECS in the HPA axis, mood disorders, and controlling negative affect (12, 19, 20, 62). While blunted stress and emotional reactivity to stressors could appear beneficial, one must consider that mounting a proper hormonal response to stress is inherently adaptive, as it permits individuals to mobilize energy stores and respond aptly to stressors in the environment (63). In the context of the current study, lower perceived stress may relate to cannabis users not mounting an appropriate response to stressors in their environment, resulting in greater problems related to their use, especially in cannabis users with a greater difficulty regulating their emotions.

Thus, it is possible that individuals with greater emotion dysregulation may be engaging in emotion-focused coping, such that they are using cannabis to effectively cope with the perceived stress and negative emotions associated with stressors (64, 65). But, because they have lowered their perceived stress, they may no longer feel the need to actively solve the stressor or problem that originally caused their perceived stress. In this way, low perceived stress could increase problematic cannabis use if individuals higher in emotion dysregulation use cannabis to decrease perceived stress and negative affect, instead of actively solving their cannabis-related problems. This would also be in line with previous studies finding emotion-focused coping to be associated with being more likely to develop and less likely to recover from substance use problems (66, 67). In contrast, the relationship between less perceived stress and more problematic cannabis use may not be as strong in individuals experiencing less emotion dysregulation because these individuals may not be as likely to engage in emotion-focused coping, since they are better able to regulate their emotions. However, these interpretations are speculative and future research, especially longitudinal and experimental designs directly examining the relationships between cannabis use and physiological and psychological reactivity to stress, are needed to elucidate the relationship between lower perceived stress and more severe problematic cannabis use in those higher in emotion dysregulation.

To our knowledge, the current study was the first to assess individual differences in emotion dysregulation as a moderator for the relationships between stressful life events and problematic cannabis use and perceived stress and problematic cannabis use. Moreover, the inclusion of both stressful life events and perceived stress allowed us to be the first, to our current knowledge, to assess their relationship with problematic cannabis use while accounting for the other. This direct comparison between different aspects of stress showed that these aspects do relate to problematic cannabis use differently, and that these relationships hold true while controlling for the other measure of stress. Furthermore, we assessed problematic cannabis use as a primary variable of interest, instead of frequency or quantity of use. Interestingly, past 30-day cannabis use was not significantly related to problematic cannabis use in any of our hierarchical multiple linear regression models, which suggests that frequency of use may not accurately reflect problematic use, and thus may not be as informative of an indicator when measuring outcomes, such as mental health.

Despite the strengths mentioned above, there are some limitations to this study. As this study is a cross-sectional, observational study, we are unable to address the directionality of potential relationships between emotion dysregulation, stress, and cannabis use. As previously mentioned, research has found that cannabis use may be associated with a dysregulated HPA axis (60); thus, it is possible that cannabis use may impact stress levels, which would oppose the current study's hypothesis of greater stress being a risk factor for problematic cannabis use. Longitudinal and experimental designs are the next step in addressing this question of causality.

Additionally, the current study only used self-report measures of stress and emotion dysregulation, which may be influenced by an individual's willingness and/or ability to accurately report on emotional responses, perceived stress, and stressful life events. The use of both behavioral and self-report measures of emotion regulation in the same study may provide a more accurate and comprehensive assessment of this complex and multi-faceted construct (68). Future studies could also consider assessing biomarkers of stress in response to a stress-evoking task in a laboratory setting to determine how physiological stress response may relate to problematic cannabis use. Finally, it is possible that there is construct overlap between the scales used for the primary variables. For example, the Holmes-Rahe Life Stress Inventory has “Fired at work” as an item, while the Marijuana Problem Scale has “To lose a job” as a problem related to cannabis use. Using more objective measures, such as behavioral measures of emotion regulation and biological measures of stress would help minimize such an issue. Thus, the results from the current study can help inform future longitudinal and experimental studies that may be interested in implementing a task-based measure of emotion regulation and physiological measures of stress.

Finally, while some recruitment took place *via* online advertising, we were unable to collect information regarding participants' state of residence. Due to our recruitment efforts in Oregon, it is likely that a majority of participants were residents from Oregon. Therefore, our results may not be generalizable to cannabis users who reside in states where cannabis has not been legalized for recreational and/or medical use. Future research may consider examining differences in these processes as a function of the legal status of the state in which data was collected.

### Conclusions

In summary, the current study found more stressful life events and greater emotion dysregulation to be associated with experiencing more severe problematic cannabis use, while less perceived stress was associated with more severe problematic cannabis use in those with greater emotion dysregulation. These findings highlight the importance of examining both emotion dysregulation and stress and comparing different aspects of stress in relation to cannabis-use outcomes. Thus, treatment and intervention efforts could benefit from focusing on teaching adaptive emotion regulation strategies and stress-management techniques to individuals seeking to reduce problems related to their cannabis use.

## Data Availability Statement

The raw data supporting the conclusions of this article will be made available by the authors, without undue reservation.

## Ethics Statement

The studies involving human participants were reviewed and approved by Oregon State University Institutional Review Board. The patients/participants provided their written informed consent to participate in this study.

## Author Contributions

JC conducted literature searches and provided summaries of previous research studies, conducted the statistical analysis, and wrote the first draft of the manuscript. AC assisted with interpretation of findings, manuscript review, and edits. All authors contributed to and have approved the final manuscript, designed the study, and wrote the protocol.

## Conflict of Interest

The authors declare that the research was conducted in the absence of any commercial or financial relationships that could be construed as a potential conflict of interest.
