# Supplementary material for: Emotion Dysregulation Moderates the Association Between Stress and Problematic Cannabis Use
Source: Front Psychiatry. 2021 Jan 8;11:597789. doi: 10.3389/fpsyt.2020.597789 (PMC7820070; doi:10.3389/fpsyt.2020.597789)
Supplement: Supplementary file 1 [file Data_Sheet_1.docx]

Supplementary Material

# Supplementary Methods

## Data Cleaning Procedures

Participants’ responses were excluded from our final analyses if they violated at least one of five rules that were established prior to any analyses. The first rule excluded any participant who completed the survey in 5 minutes or less. Wood et al. (2017) recommend that in online samples, researchers remove participants who complete survey questions at rates faster than 1 second per item (i.e. for our survey, that would mean removing participants who completed it in 145 seconds or less). Though, based on average completion time (40 minutes) by research assistants, we decided to take a more conservative approach, and exclude anyone who completed the survey in 5 minutes or less. This resulted in 119 of 1,586 participants being excluded. Our second rule excluded anyone who reported no lifetime cannabis use, which resulted in 367 participants being excluded. Our third rule excluded anyone who reported different ages when asked about their current age at two different points during the survey; 71 participants broke this rule. Our fourth rule excluded anyone who reported their past-day 30 cannabis use to be greater than their lifetime cannabis use; 12 participants broke this rule. Finally, our fifth rule excluded any participants who reported that they either: 1) did use cannabis in the past six months on the Cannabis Use Disorder Identification Test – Revised (CUDIT-R; Adamson & Sellman, 2003), but reported their last cannabis use was more than one year ago on the Daily Sessions, Frequency, Age of Onset, and Quantity of Cannabis Use Inventory (DFAQ-CU; Cuttler & Spradlin, 2017), or 2) did not use cannabis in the past six months on the CUDIT-R, but indicated their last cannabis use was less than one month ago on the DFAQ-CU; 129 participants broke this rule.

# Supplementary Results

Like Model 1, regression Model 3 tested whether stressful life events, emotion dysregulation, and their interaction predicted problematic cannabis use, though more covariates were added. In addition to demographic variables and past 30-day cannabis use, we also added past 30-day alcoholic drinks and e-nicotine use as covariates and found a significant regression equation (*F*(31,820) = 40.59, *p* <.001) with *R^2^* = .61 (**Supplementary Table 1**). Emotion dysregulation (*B* = .40, *p* < .001), stressful life events (*B* = .21, *p* <.001) and their interaction (*B* = .06, *p* = .01) were significant predictors of problematic cannabis use (**Supplementary Table 2**).

Like Model 2, regression Model 4 tested whether perceived stress, emotion dysregulation, and their interaction predicted problematic cannabis use, though more covariates were added. In addition to demographic variables and past 30-day cannabis use, we also added past 30-day alcoholic drinks and e-nicotine use as covariates and found a significant regression equation (*F*(31,820) = 40.45, *p* <.001) with *R^2^* = .61 (**Supplementary Table 3**). Emotion dysregulation (*B* = .39, *p* < .001), perceived stress (*B* = -.18 *p* <.001), and their interaction (*B* = -.06, *p* = .03) were significant predictors of problematic cannabis use (**Supplementary Table 4**).

**3 References**

Adamson, S. J., & Sellman, J. D. (2003). A prototype screening instrument for cannabis use disorder: The Cannabis Use Disorders Identification Test (CUDIT) in an alcohol-dependent clinical sample. *Drug and Alcohol Review*, *22*(3), 309–315. https://doi.org/10.1080/0959523031000154454

Cuttler, C., & Spradlin, A. (2017). Measuring cannabis consumption: Psychometric properties of the Daily Sessions, Frequency, Age of Onset, and Quantity of Cannabis Use Inventory (DFAQ-CU). *PLoS ONE*, *12*(5). https://doi.org/10.1371/journal.pone.0178194

Wood, D., Harms, P. D., Lowman, G. H., & DeSimone, J. A. (2017). Response Speed and Response Consistency as Mutually Validating Indicators of Data Quality in Online Samples. *Social Psychological and Personality Science*, *8*(4), 454–464. https://doi.org/10.1177/1948550617703168

# Supplementary Tables

| **Model Summary** | | | | | | | | | | |
| --- | --- | --- | --- | --- | --- | --- | --- | --- | --- | --- |
|  | R | R^2^ | Adj. R^2^ | Std. Error of the Estimate | R^2^ Change | *F* Change | df1 | df2 | Sig. F Change | Cohen's f^2^ |
| Block 1 | 0.68 | 0.46 | 0.44 | 7.60 | 0.46 | 24.88 | 28 | 823 | **<.001** |  |
| Block 2 | 0.78 | 0.60 | 0.59 | 6.50 | 0.14 | 148.44 | 2 | 821 | **<.001** | 0.35 |
| Block 3 | 0.78 | 0.61 | 0.59 | 6.47 | 0.003 | 6.62 | 1 | 820 | **0.01** | 0.03 |

**Supplementary Table 1.** Model summary for model 3: Effects of emotion dysregulation, stressful life events, and their interaction on problematic cannabis use, with substance use covariates.

| **Coefficients** | | | | | | | | | | | |  | |  | |
| --- | --- | --- | --- | --- | --- | --- | --- | --- | --- | --- | --- | --- | --- | --- | --- |
|  |  | Unstandardized | | Standardized |  |  | 95% CI for B | | Correlations | | |  |  | |  |
|  |  | B | Std. Error | Beta | *t* | Sig. | Lower Bound | Upper Bound | Zero-order | Partial | Part |  |  | |  |
| Block 1 | (Constant) | 6.88 | 3.19 |  | 2.16 | **0.03** | 0.63 | 13.14 |  |  |  |  |  | |  |
|  | Age | 0.05 | 0.04 | 0.04 | 1.33 | 0.18 | -0.03 | 0.14 | 0.29 | 0.05 | 0.03 |  |  | |  |
|  | Sex | -2.35 | 0.53 | -0.11 | -4.45 | **< .001** | -3.39 | -1.31 | -0.39 | -0.15 | -0.1 |  |  | |  |
|  | Hispanic/Latinx | 2.36 | 0.55 | 0.11 | 4.31 | **< .001** | 1.28 | 3.43 | 0.28 | 0.15 | 0.09 |  |  | |  |
|  | Unknown (Hispanic/Latinx) | 0.21 | 2.27 | 0 | 0.09 | 0.93 | -4.24 | 4.66 | -0.05 | 0 | 0 |  |  | |  |
|  | White | 0.63 | 2.19 | 0.03 | 0.29 | 0.78 | -3.67 | 4.93 | -0.08 | 0.01 | 0.01 |  |  | |  |
|  | Black or African American | 3.22 | 2.28 | 0.13 | 1.42 | 0.16 | -1.25 | 7.69 | 0.22 | 0.05 | 0.03 |  |  | |  |
|  | American Indian or Alaskan Native | 5.07 | 2.46 | 0.1 | 2.06 | **0.04** | 0.23 | 9.9 | 0.08 | 0.07 | 0.05 |  |  | |  |
|  | Asian | -3.63 | 2.5 | -0.07 | -1.46 | 0.15 | -8.53 | 1.27 | -0.18 | -0.05 | -0.03 |  |  | |  |
|  | More than One Race | -2.55 | 2.47 | -0.05 | -1.03 | 0.3 | -7.39 | 2.3 | -0.17 | -0.04 | -0.02 |  |  | |  |
|  | Native Hawaiian/Other Pacific Islander | 5.01 | 2.77 | 0.07 | 1.81 | 0.07 | -0.43 | 10.45 | 0.07 | 0.06 | 0.04 |  |  | |  |
|  | Unknown (Race) | -3.69 | 5.07 | -0.02 | -0.73 | 0.47 | -13.64 | 6.25 | -0.06 | -0.03 | -0.02 |  |  | |  |
|  | Some High School | 5.65 | 4.2 | 0.03 | 1.35 | 0.18 | -2.59 | 13.89 | 0.03 | 0.05 | 0.03 |  |  | |  |
|  | High School Diploma/GED | -1.32 | 1.95 | -0.03 | -0.68 | 0.5 | -5.15 | 2.51 | -0.18 | -0.02 | -0.01 |  |  | |  |
|  | Trade/Technical/Vocational Training | -0.04 | 1.89 | 0 | -0.02 | 0.98 | -3.74 | 3.66 | 0.03 | 0 | 0 |  |  | |  |
|  | Some College | -0.86 | 1.73 | -0.04 | -0.5 | 0.62 | -4.26 | 2.54 | -0.34 | -0.02 | -0.01 |  |  | |  |
|  | Associate's Degree | 0.62 | 1.74 | 0.02 | 0.36 | 0.72 | -2.8 | 4.04 | -0.01 | 0.01 | 0.01 |  |  | |  |
|  | Bachelor's Degree | 4.25 | 1.71 | 0.19 | 2.48 | **0.01** | 0.89 | 7.61 | 0.42 | 0.09 | 0.05 |  |  | |  |
|  | Some Graduate School | 2.35 | 1.94 | 0.05 | 1.21 | 0.23 | -1.46 | 6.16 | 0.09 | 0.04 | 0.03 |  |  | |  |
|  | $0 | 1.11 | 2.32 | 0.01 | 0.48 | 0.63 | -3.43 | 5.66 | -0.12 | 0.02 | 0.01 |  |  | |  |
|  | $0-$5,000 | -0.75 | 1.54 | -0.01 | -0.49 | 0.63 | -3.77 | 2.27 | -0.16 | -0.02 | -0.01 |  |  | |  |
|  | $5,000-$10,000 | 1.19 | 1.19 | 0.03 | 1 | 0.32 | -1.14 | 3.51 | -0.19 | 0.03 | 0.02 |  |  | |  |
|  | $10,000-$50,000 | 3.78 | 0.9 | 0.14 | 4.23 | **< .001** | 2.03 | 5.54 | -0.05 | 0.15 | 0.09 |  |  | |  |
|  | $50,000-$75,000 | 4.36 | 0.79 | 0.21 | 5.49 | **< .001** | 2.8 | 5.92 | 0.36 | 0.19 | 0.12 |  |  | |  |
|  | $75,000-$100,000 | 1.06 | 0.86 | 0.04 | 1.24 | 0.22 | -0.62 | 2.74 | -0.02 | 0.04 | 0.03 |  |  | |  |
|  | Cannabis Use Days^a^ | -0.01 | 0.03 | -0.01 | -0.46 | 0.65 | -0.07 | 0.04 | 0.08 | -0.02 | -0.01 |  |  | |  |
|  | Alcoholic Drinks^b^ | 0.02 | 0.05 | 0.01 | 0.5 | **0.002** | -0.07 | 0.12 | -0.17 | 0.02 | 0.01 |  |  | |  |
|  | E-Nicotine Use Days^c^ | -0.05 | 0.02 | -0.08 | -3.11 | .62 | -0.09 | -0.02 | -0.31 | -0.11 | -0.07 |  |  | |  |
|  | Centered PSS Scores | -0.26 | 0.05 | -0.15 | -5.44 | **< .001** | -0.36 | -0.17 | 0.13 | -0.19 | -0.12 |  |  | |  |
| Block 2 | Centered DERS Scores | 0.2 | 0.02 | 0.4 | 13.38 | **< .001** | 0.17 | 0.23 | 0.53 | 0.42 | 0.29 |  |  | |  |
|  | Centered H-RLSI Scores | 0.02 | 0 | 0.21 | 8.52 | **< .001** | 0.01 | 0.02 | 0.32 | 0.29 | 0.19 |  |  | |  |
| Block 3 | DERS X H-RLSI | 0 | 0 | 0.06 | 2.57 | **0.01** | 0 | 0 | 0.15 | 0.09 | 0.06 |  |  | |  |

**Supplementary Table 2.** Coefficients for model 3: Effects of emotion dysregulation, stressful life events, and their interaction on problematic cannabis use, with substance use covariates. *Note*. PSS = Perceived Stress Scale. DERS = Difficulties in Emotion Regulation Scale. H-RLSI =Holmes-Rahe Life Stress Inventory. ^a^Number of days cannabis was used in the past 30 days. ^b^Number of drinks consumed in the past 30 days. ^c^Number of days e-nicotine products were used in the past 30 days.

| **Model Summary** | | | | | | | | | | |
| --- | --- | --- | --- | --- | --- | --- | --- | --- | --- | --- |
|  | R | R^2^ | Adj. R^2^ | Std. Error of the Estimate | R^2^ Change | *F* Change | df1 | df2 | Sig. F Change | Cohen's f^2^ |
| Block 1 | 0.72 | 0.51 | 0.50 | 7.20 | 0.51 | 31.05 | 28 | 823 | **<.001** |  |
| Block 2 | 0.78 | 0.60 | 0.59 | 6.50 | 0.09 | 91.40 | 2 | 821 | **<.001** | 0.23 |
| Block 3 | 0.78 | 0.60 | 0.59 | 6.48 | 0.002 | 4.90 | 1 | 820 | **0.03** | 0.008 |

**Supplementary Table 3.** Model summary for model 4: effects of emotion dysregulation, perceived stress, and their interaction on problematic cannabis use, with substance use covariates.

| **Coefficients** | | | | | | | | | | | |  | |  | |
| --- | --- | --- | --- | --- | --- | --- | --- | --- | --- | --- | --- | --- | --- | --- | --- |
|  |  | Unstandardized | | Standardized |  |  | 95% CI for B | | Correlations | | |  |  | |  |
|  |  | B | Std. Error | Beta | *t* | Sig. | Lower Bound | Upper Bound | Zero-order | Partial | Part |  |  | |  |
| Block 1 | (Constant) | 7.05 | 3.19 |  | 2.21 | **0.03** | 0.79 | 13.3 |  |  |  |  |  | |  |
|  | Age | 0.07 | 0.04 | 0.04 | 1.63 | 0.1 | -0.01 | 0.15 | 0.29 | 0.06 | 0.04 |  |  | |  |
|  | Sex | -2.19 | 0.53 | -0.1 | -4.11 | **< .001** | -3.23 | -1.14 | -0.39 | -0.14 | -0.09 |  |  | |  |
|  | Hispanic/Latinx | 2.32 | 0.55 | 0.11 | 4.24 | **< .001** | 1.25 | 3.4 | 0.28 | 0.15 | 0.09 |  |  | |  |
|  | Unknown (Hispanic/Latinx) | 0.35 | 2.27 | 0 | 0.15 | 0.88 | -4.1 | 4.8 | -0.05 | 0.01 | 0 |  |  | |  |
|  | White | 0.56 | 2.19 | 0.03 | 0.26 | 0.8 | -3.74 | 4.87 | -0.08 | 0.01 | 0.01 |  |  | |  |
|  | Black or African American | 3.18 | 2.28 | 0.12 | 1.4 | 0.16 | -1.29 | 7.66 | 0.22 | 0.05 | 0.03 |  |  | |  |
|  | American Indian or Alaskan Native | 4.98 | 2.47 | 0.1 | 2.02 | **0.04** | 0.14 | 9.82 | 0.08 | 0.07 | 0.04 |  |  | |  |
|  | Asian | -3.73 | 2.5 | -0.07 | -1.49 | 0.14 | -8.63 | 1.17 | -0.18 | -0.05 | -0.03 |  |  | |  |
|  | More than One Race | -2.89 | 2.46 | -0.05 | -1.18 | 0.24 | -7.72 | 1.93 | -0.17 | -0.04 | -0.03 |  |  | |  |
|  | Native Hawaiian/Other Pacific Islander | 5.06 | 2.77 | 0.07 | 1.82 | 0.07 | -0.38 | 10.51 | 0.07 | 0.06 | 0.04 |  |  | |  |
|  | Unknown (Race) | -2.93 | 5.07 | -0.01 | -0.58 | 0.56 | -12.89 | 7.02 | -0.06 | -0.02 | -0.01 |  |  | |  |
|  | Some High School | 4.95 | 4.2 | 0.03 | 1.18 | 0.24 | -3.31 | 13.2 | 0.03 | 0.04 | 0.03 |  |  | |  |
|  | High School Diploma/GED | -1.28 | 1.95 | -0.03 | -0.66 | 0.51 | -5.11 | 2.55 | -0.18 | -0.02 | -0.01 |  |  | |  |
|  | Trade/Technical/Vocational Training | -0.42 | 1.89 | -0.01 | -0.22 | 0.82 | -4.14 | 3.3 | 0.03 | -0.01 | 0 |  |  | |  |
|  | Some College | -1.04 | 1.74 | -0.05 | -0.6 | 0.55 | -4.44 | 2.37 | -0.34 | -0.02 | -0.01 |  |  | |  |
|  | Associate's Degree | 0.56 | 1.74 | 0.02 | 0.32 | 0.75 | -2.87 | 3.98 | -0.01 | 0.01 | 0.01 |  |  | |  |
|  | Bachelor's Degree | 4.03 | 1.72 | 0.18 | 2.35 | **0.02** | 0.66 | 7.4 | 0.42 | 0.08 | 0.05 |  |  | |  |
|  | Some Graduate School | 2.16 | 1.95 | 0.05 | 1.11 | 0.27 | -1.66 | 5.98 | 0.09 | 0.04 | 0.02 |  |  | |  |
|  | $0 | 1.08 | 2.32 | 0.01 | 0.46 | 0.64 | -3.48 | 5.63 | -0.12 | 0.02 | 0.01 |  |  | |  |
|  | $0-$5,000 | -0.66 | 1.54 | -0.01 | -0.43 | 0.67 | -3.68 | 2.36 | -0.16 | -0.01 | -0.01 |  |  | |  |
|  | $5,000-$10,000 | 0.92 | 1.18 | 0.02 | 0.78 | 0.44 | -1.4 | 3.24 | -0.19 | 0.03 | 0.02 |  |  | |  |
|  | $10,000-$50,000 | 3.59 | 0.9 | 0.13 | 4.01 | **< .001** | 1.83 | 5.35 | -0.05 | 0.14 | 0.09 |  |  | |  |
|  | $50,000-$75,000 | 4.25 | 0.8 | 0.21 | 5.34 | **< .001** | 2.69 | 5.81 | 0.36 | 0.18 | 0.12 |  |  | |  |
|  | $75,000-$100,000 | 1.2 | 0.86 | 0.05 | 1.4 | 0.16 | -0.48 | 2.88 | -0.02 | 0.05 | 0.03 |  |  | |  |
|  | Cannabis Use Days^a^ | -0.01 | 0.03 | -0.01 | -0.32 | 0.75 | -0.06 | 0.05 | 0.08 | -0.01 | -0.01 |  |  | |  |
|  | Alcoholic Drinks^b^ | -0.05 | 0.02 | -0.07 | -2.76 | **0.01** | -0.08 | -0.01 | -0.31 | -0.1 | -0.06 |  |  | |  |
|  | E-Nicotine Use Days^c^ | 0.01 | 0.05 | 0.01 | 0.28 | 0.78 | -0.08 | 0.11 | -0.17 | 0.01 | 0.01 |  |  | |  |
|  | Centered H-RLSI Scores | 0.02 | 0 | 0.22 | 8.92 | **< .001** | 0.02 | 0.02 | 0.32 | 0.3 | 0.2 |  |  | |  |
| Block 2 | Centered DERS Scores | 0.2 | 0.02 | 0.39 | 13.18 | **< . 001** | 0.17 | 0.23 | 0.53 | 0.42 | 0.29 |  |  | |  |
|  | Centered PSS Scores | -0.32 | 0.05 | -0.18 | -6.07 | **< .001** | -0.43 | -0.22 | 0.13 | -0.21 | -0.13 |  |  | |  |
| Block 3 | DERS X PSS | 0 | 0 | -0.06 | -2.21 | **0.03** | -0.01 | 0 | -0.26 | -0.08 | -0.05 |  |  | |  |

**Supplementary Table 4.** Coefficients for model 4: Effects of emotion dysregulation, perceived stress, and their interaction on problematic cannabis use, with substance use covariates. *Note*. H-RLSI =Holmes-Rahe Life Stress Inventory. DERS = Difficulties in Emotion Regulation Scale. PSS = Perceived Stress Scale. ^a^Number of days cannabis was used in the past 30 days. ^b^Number of drinks consumed in the past 30 days. ^c^Number of days e-nicotine products were used in the past 30 days.
